# Effects of multiple stressors in fish: how parasites and contaminants interact

**DOI:** 10.1017/S0031182022001172

**Published:** 2022-12

**Authors:** Bernd Sures, Milen Nachev

**Affiliations:** Department of Aquatic Ecology and Centre for Water and Environmental Research (ZWU), University of Duisburg-Essen, Essen, Germany

**Keywords:** Aquatic parasites, Environmental Parasitology, pollution

## Abstract

Interest in local environmental conditions and the occurrence and behaviour of parasites has increased over the last 3 decades, leading to the discipline of Environmental Parasitology. The aim of this discipline is to investigate how anthropogenically altered environmental factors influence the occurrence of parasites and how the combined effects of pollutants and parasites affect the health of their hosts. Accordingly, in this paper, we provide an overview of the direct and indirect effects of pollutants on the occurrence and distribution of fish parasites. However, based on current knowledge, it is difficult to draw general conclusions about these interdependencies, as the effects of pollutants on free-living (larval) parasite stages, as well as their effects on ectoparasites, depend on the pollutant–host–parasite combination as well as on other environmental factors that can modulate the harmful effects of pollutants. Furthermore, the question of the combined effects of the simultaneous occurrence of parasites and pollutants on the physiology and health of the fish hosts is of interest. For this purpose, we differentiate between the dominance effects of individual stressors over other, additive or synergistically reinforcing effects as well as combined antagonistic effects. For the latter, there are only very few studies, most of which were also carried out on invertebrates, so that this field of research presents itself as very promising for future investigations.

## Graphical abstract

**Figure:**
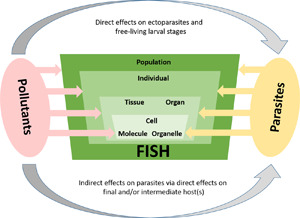

## Introduction

Aquatic organisms, and fish in particular, are affected by a variety of stressors caused by anthropogenic influences that lead to changes in environmental parameters. These in turn elicit stress responses of the organism in the sense that the affected organisms show reactions outside their normal range. Stressors include chemical pollutants (i.e. contaminants), nutrients, flow velocity, pH, dissolved oxygen, disturbance in light and temperature regimes and many other physico-chemical variables that can be significantly altered by anthropogenic activities (e.g. Birk *et al*., 2020). In this paper, the focus is mainly on the chemical pollutants that can cause acute and/or chronic effects in biota when exceeding aqueous concentrations above their natural range of occurrence. However, other factors such as the temperature regime, pH and dissolved oxygen are also considered, as they may influence the solubility and bioavailability of pollutants or, conversely, the pollutants may influence the natural ranges of some of these factors (e.g. oxygen content, pH) and thus contribute significantly to the effects of the pollutants.

Chemical contaminants include various micropollutants of inorganic and organic nature, organometallic compounds, but also dissolved salts (NaCl, CO_3_^2−^) and nutrients (e.g. NO_3_^−^, NO_2_^−^, NH_4_^+^, PO_4_^3−^). The inorganic pollutants comprise various trace elements (metals and metalloids such as Cd, Pb, As), which may be of geogenic origin (Erasmus *et al*., 2022). In most cases, however, the elevated concentrations are related to anthropogenic activities such as mining, industrial and domestic wastewater, agriculture, erosion of landfills, waste dump and many others (e.g. Schertzinger *et al*., 2018, 2019; Kontchou *et al*., 2021; Rothe *et al*., 2021; Erasmus *et al*., 2022; Link *et al*., 2022). Metals can affect for example the embryonic and larval development of fish, its growth and fitness as well as reproduction (e.g. summarized in Taslima *et al*., 2022). The modes of action comprise effects on molecular and cellular levels as well as on the immune system, on the physiology, and the metabolism of fish (see Taslima *et al*. (2022) and references therein). Some metals such as Cd, Cr, Hg and Pb may also act as endocrine disruptors (reviewed in Chakraborty, 2021).

Elevated levels of dissolved salts (e.g. salinization) and nutrients are also related to anthropogenic activities and can directly affect fish or indirectly affect environmental conditions (e.g. in the case of eutrophication) and the species composition of the biota (Schröder *et al*., 2015) as well as food availability for fish in general. Organic pollutants include a large group of compounds used in industry (e.g. PCBs, PAHs, plasticizers, flame retardants) and agriculture (e.g. pesticides), but also some that are used as pharmaceuticals or personal care products or enter domestic wastewater as metabolic end products. Similar to inorganic pollutants, they can have effects on different levels of organization in fish and might additionally act as endocrine disruptors (Tierney *et al*., 2013).

In addition to the pollutants mentioned, parasites might represent an additional stressor for fish at the individual, population or community level. Parasite infections can reduce host fitness by inducing different pathological, immunological and physiological responses (e.g. Sures *et al*., 2001; Münderle *et al*., 2004; Buchmann and Bresciani, 2006; Gérard *et al*., 2016). Nutrition and energy drain as well as host manipulation can reduce the performance and fitness of infected individuals. Parasite infection can also negatively affect the dynamics and density of fish populations and thus the entire fish community. Population effects are particularly significant for infections with parasites that cause severe pathological damage (e.g. Sokolowski and Dove, 2006; Shafaquat *et al*., 2016; Barišić *et al*., 2018; Dezfuli *et al*., 2021) or with parasites that act as endocrine disruptors and parasitic castrators (e.g. Trubiroha *et al*., 2010, 2011) and/or manipulate their host to make it more vulnerable to predation (e.g. Giles, 1983; Museth, 2001; Gabagambi *et al*., 2019; Svensson *et al*., 2022).

**Fig. 1.**
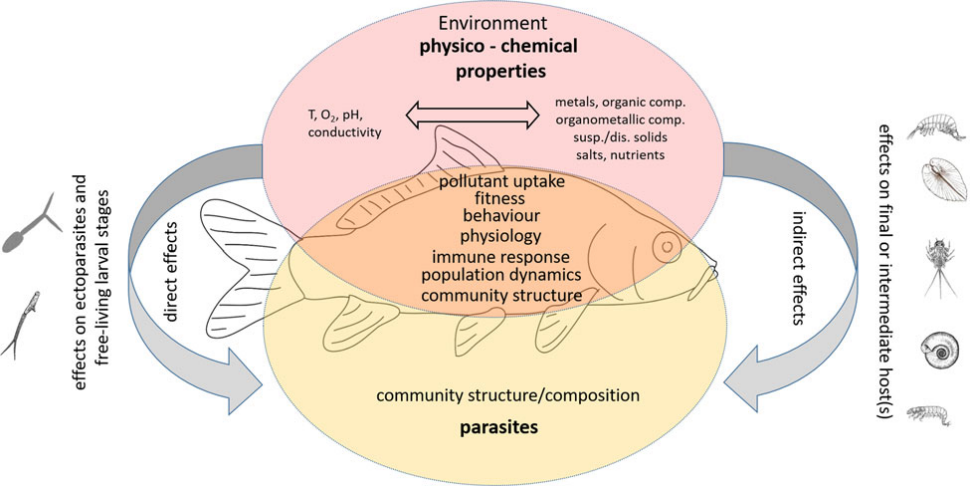
Changes in pollution levels of aquatic habitats can **directly** or **indirectly** affect the structure and composition of fish parasite communities. The direct mode of action includes mainly effects on adult ectoparasites or larval stages that are in immediate contact with the environment. Adverse effects of pollutants lead to lower transmission efficiency of parasites, which in turn affects the structure and dynamics of parasite populations.

In general, parasite infections increase the susceptibility of their hosts to various stressors (Combes, 1995), so that infected fish exposed to multiple stressors may react differently than uninfected conspecifics. The aim of this paper is therefore to summarize and discuss what is known about the complex interactions between parasites and various contaminants from the perspective of the fish host (see Fig. 1). In terms of Environmental Parasitology, this overview sheds light on the role of parasites in ecosystems where multiple stressors interact. First, we provide an overview of (i) the direct interaction between parasite stages which are in close contact to environmental contaminants (i.e. ectoparasites and free-living stages of endoparasites), before (ii) summarizing indirect effects of pollutants on fish parasite occurrence and distribution. Additionally, we give examples of (iii) the complex fish–parasite–pollution interaction and finally, provide some (iv) concluding remarks and an outlook for future research.

## Direct interaction between parasites and contaminants in the aquatic environment

Parasites constitute a significant part of natural ecosystems and produce a considerable amount of their biomass (Kuris *et al*., 2008; Soldánová *et al*., 2016). Similar to free-living organisms, parasites are affected by and respond to environmental conditions. Fluctuations in the population dynamics of parasites are still not very well understood, but in several cases, the reasons lie in changes in environmental conditions (pollution, anthropogenic impact, etc.; see Thieltges *et al*., 2008) as well as in invasive species encountering susceptible hosts after their arrival and then succeeding accordingly (Goedknegt *et al*., 2016). Over the last 2 decades, research has shown that environmental factors have a significant impact on parasites and can directly influence the composition and diversity of parasite communities. The direct mode of action includes effects on adult ectoparasites or larval stages that are in immediate contact with the environment (Gheorgiu *et al*., 2006; Gheorghiu *et al*., 2007; Thieltges *et al*., 2008; Sures *et al*., 2017). Pollution-induced lethal responses lead to lower transmission efficiency of parasites, which in turn affects the structure and dynamics of parasite populations. Moreover, ectoparasites have been found to respond very sensitive to pollution (e.g. eutrophication or chemical pollution) (Gilbert and Avenant-Oldewage, 2021) similar to endoparasites with free-living larval stages (e.g. cercariae, miracidia), which may also be directly affected by environmental conditions (summarized by Thieltges *et al*., 2008). A meta-analysis of published data (Gilbert and Avenant-Oldewage, 2021) has shown that pollution can have both positive and negative effects on monogenean communities, while some pollutants show no clear effects. It can be seen that metals tend to have a negative effect on monogenean communities (lower abundance of some species and lower diversity), while eutrophication has a positive effect (see Gilbert and Avenant-Oldewage, 2021 and references therein). Direct effects of pollutants on endoparasites are due to exposure of free-living larval stages to pollutants. Metals have been reported to affect trematode transmission by reducing the longevity, viability and infectivity of cercariae (Pietrock and Marcogliese, 2003; Morley *et al*., 2006). Similar effects were reported for organic pollutants such as various pesticides (e.g. Koprivnikar *et al*., 2006, 2007; Rohr *et al*., 2008; Raffel *et al*., 2009; Hua *et al*., 2016).

Free-living stages of endoparasites and ectoparasites can not only be affected by pollutants, but also should in turn be able to influence the level of pollutants in the environment. For example, cercariae, which are excreted in large numbers and biomass by the molluscan intermediate host (Soldánová *et al*., 2016; Díaz-Morales *et al*., 2022), could act as a pollutant sink at low spatial scales, influencing the distribution and further bioavailability of chemicals when accumulated by them. During their short lifespan, cercariae provide a food source for various aquatic organisms (Johnson *et al*., 2010), including fish, and thus may additionally contribute to the distribution and translocation of chemicals within the various components of food webs. It can be assumed that a significant proportion of pollutants could be stored in free-living stages of parasites in ecosystems, as various parasitic taxa have an excellent accumulation capacity (see e.g. Sures *et al*., 2017). Interestingly, to the best of our knowledge, no study is available addressing a possible pollutant uptake by cercariae although studies by Morley and colleagues (Morley *et al*., 2003, 2006) suggest the availability of metals for cercariae with subsequent fatal effects. Regardless of a possible pollutant load, the majority of the cercariae die, sediment together with the suspended matter and become demineralized. Ectoparasites such as crustaceans and monogeneans, which can accumulate metals (Pérez-del-Olmo *et al*., 2019; Nachev *et al*., 2022), can also presumably take up pollutants from the environment. It has already been shown that, for example, metals may become incorporated into the sclerotized structures of the haptor in monogeneans (Gilbert and Avenant-Oldewage, 2017). Often, such a contaminant exposure lead to malformation of the haptor in several families of monogeneans (see e.g. Šebelová *et al*., 2002; Pečínková *et al*., 2005; Dzika *et al*., 2007; Rodríguez-González *et al*., 2020) and could therefore even be used to indicate metal pollution in aquatic ecosystems (Gilbert and Avenant-Oldewage, 2021).

## Indirect effects of contaminants on parasite occurrence and distribution in the aquatic environment

Indirect effects of contaminants on individual parasites and their communities refer to the presence and abundance of free-living intermediate or definitive hosts involved in the life cycle of multi-host parasites. Host organisms require an optimal range of environmental conditions and respond to deviations from these or to the presence of stressors with reduced abundance, while in the extreme range of conditions they may even be absent. As a result, parasites show lower species richness and diversity, and changes in species composition. Nachev and Sures (2009) reported lower parasite diversity in fish from polluted sites (higher metal concentrations and eutrophication) in comparison to less polluted localities. Similar patterns were reported by Krause *et al*. (2010) in relation to general water quality in combination with adverse environmental conditions as well as by Barišić *et al*. (2018) and Braicovich *et al*. (2020) as a consequence of industrial and agricultural activities and effluent of a wastewater treatment plant. The richness and structure of parasite communities were found to follow gradients of salinity and eutrophication, with fecal coliform counts and temperature serving as proxies for the pulp mill and municipal effluents (Blanar *et al*., 2011), land use and the concentration of hydrocarbons (PAHs) in sediments (Blanar *et al*., 2016), pharmaceuticals (Pravdová *et al*., 2022) or levels in PCBs in sediments (Carreras-Aubets *et al*., 2012) as well as the level of urbanization (Taglioretti *et al*., 2018). Also anthropogenic activities such as clearcutting (Marcogliese *et al*., 2001) and precipitation (Marcogliese *et al*., 2016), which alter the temperature, chemistry and hydrology of aquatic systems and the composition of free-living communities, were found to impact the communities of fish parasites.

But also the opposite scenario has been described in the literature. The host's physiology and immune system may undergo a number of stressor-induced changes when exposed to environmental contaminants that are beneficial to the parasites' infection success, for example, if the host's immune system is weakened. In this case, levels of pollutants positively correlate with the abundance of some parasite taxa (Marcogliese, 2004, 2005). Positive effects are described for ectoparasites such as monogeneans, which often occur in higher abundance and diversity as a result of the host's immune system being compromised by pollution (Pérez-del Olmo *et al*., 2007; Sanchez-Ramirez *et al*., 2007; Pravdová *et al*., 2021). Although comparatively, much information is available on the effects of contaminants on the immune system of fish, most of these studies are correlative in nature. Mechanistic studies, on the other hand, dealing with the immunotoxicity of xenobiotics are mainly based on challenges by viral and bacterial pathogens or synthetic antigens (Regala *et al*., 2001; Sures, 2008*a*). Comparable studies investigating the immunosuppressive effects of environmental pollutants on metazoan parasites are relatively rare, as they are quite complicated to conduct (Hoole, 1997). Laboratory infection experiments of European eels (*Anguilla anguilla*) with the swim bladder nematode *Anguillicola crassus* revealed that exposure of eels to PCB 126 resulted in a complete suppression of the eels' antibody response although no higher infection intensities were found in PCB 126-exposed eels when compared to unexposed conspecifics (Sures and Knopf, 2004). Moreover, a combined Cd- and PCB 126-exposure together with experimental infection with *A. crassus* induced significantly increased cortisol levels in eel blood, which themselves are assumed to be immunosuppressive (Sures *et al*., 2006).

## Interactive effects of simultaneously occurring pollution and parasitism on fish

**Fig. 2.**
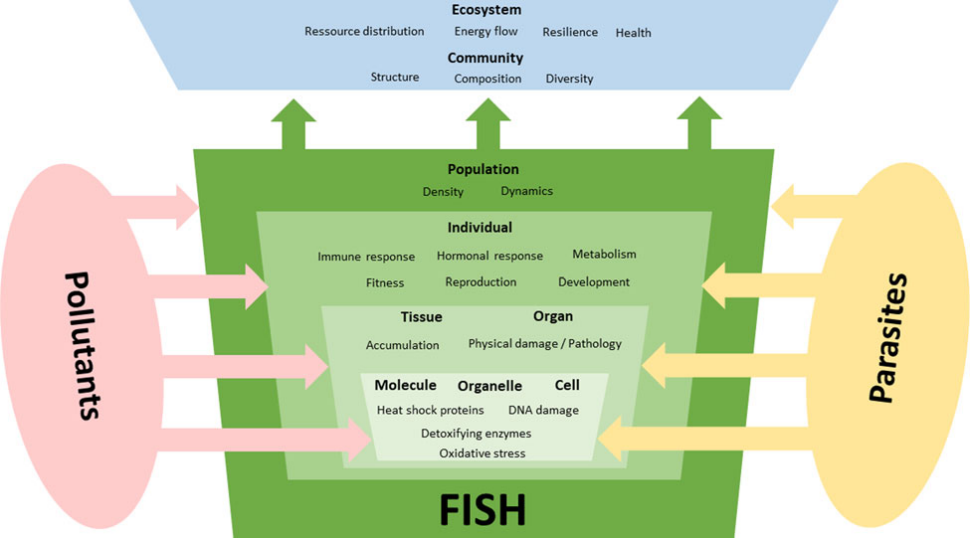
Interactive effects of pollutants and parasites on different organization levels of fish. Parasites are an additional stressor for fish that might superimpose the effects of environmental factors, which can lead to various forms of **stressor interaction**. In addition to frequently observed **additive** and **synergistic** negative effects on fish, there are also examples of **antagonistic** effects where parasite infections appear to be beneficial to infected individuals. Also, **dominance effects** might occur where 1 stressor outweigh effects of the other stressor. Effects of these stressors often manifest on molecular and subcellular levels but their effects might be seen on the population or even community level.

Following the ecological concept of stressor interaction (Birk *et al*., 2020) pollutants and parasites can interact in many ways (Fig. 2) and 3 main types of impact on their hosts can be distinguished: (1) only 1 of the 2 stressors has relevant effects on the host, i.e. the effects of 1 stressor outweigh those of the other stressor (stressor dominance); (2) parasites and pollutants act independently in a way that their joint effect is the sum of the individual effects (additive effects); and (3) 1 stressor either strengthens (synergistic) or weakens (antagonistic) the effects of the other. As previously mentioned various organic and inorganic contaminants can act as toxic substances with adverse effects on the physiology of the host that can be modulated by simultaneously occurring parasites.

In the case of a dominant stressor, there are examples of both parasites as well as pollutants. Especially monoxenous parasites with short life cycles and high infectivity can quickly threaten fish to such an extent that they die, so that further stressors such as pollutants hardly play a role in these scenarios. For example, the monogenean *Gyrodactylus salaris* has shown high virulence towards East Atlantic salmon in rivers in Norway, leading to high mortality of fry and parr (Heinecke *et al*., 2007 and references therein). On the other hand, pollutants can also show stressor dominance, especially if they are really highly toxic substances that are present in correspondingly high concentrations. There are a number of environmental disasters that show how dominant and fatal pollution as a stressor can be. One of the most prominent ones in freshwater ecosystems is probably the Sandoz accident that occurred in 1986 in Switzerland (Van Urk *et al*., 1993). A fire in a chemical production plant led to the release of toxic agrochemicals into the River Rhine, killing a large part of the eel population and severely damaging other fish species and macroinvertebrates as far downstream as the Netherlands (Güttinger and Stumm, 1992).

Probably, the most common scenario is where the negative effects of pollutants and parasites are more pronounced when they occur together, in the sense of an additive or synergistic interaction, than if only a single stressor were present (Fig. 2). Accordingly, in these cases, the damage to the fish is also significantly greater as if only a single stressor occurs (Sures, 2008*b*). Many studies have shown that acute and chronic effects of chemicals can be exacerbated by parasitism, through lower fish survival (e.g. Boyce and Yamada, 1977; Pascoe and Cram, 1977; Gheorgiu *et al*., 2006), poorer body condition (e.g. Thilakaratne *et al*., 2007) and various physiological markers (e.g. Marcogliese and Pietrock, 2011 and references therein; Sures *et al*., 2017 and references therein). In particular, interest in the interaction between parasites and environmental pollution has increased in recent years in terms of biomarker responses. Currently, it appears that mostly the pathogenicity of parasites can be enhanced when they coexist with pollutants, such that the negative effects induced by pollution are exacerbated by the parasites (Marcogliese *et al*., 2010; Frank *et al*., 2013). However, the modulation of pollutant–biomarker responses in organisms by parasites is a phenomenon that is currently not well understood and therefore deserves further investigation (Sures *et al*., 2017).

The most interesting interactions are antagonistic effects, where the presence of 1 stressor mitigates the negative effects of the other. The ability of some helminth species such as cestodes, acanthocephalans and nematodes to accumulate pollutants in high concentrations is the best-known example in this context. Due to their enormous pollutant (mainly metals) accumulation capacity, these parasites can significantly reduce the levels of accumulated pollutants within the fish body (summarized in Sures *et al*., 2017). This has been demonstrated in laboratory and field studies for concentrations of various trace elements whose concentrations were reduced in fish infected by acanthocephalans (Sures *et al*., 1999, 2003; Filipović Marijić *et al*., 2013, 2014; Brázová *et al*., 2015; Paller *et al*., 2016), cestodes (Gabrashanska and Nedeva, 1996; Turcekova and Hanzelova, 1999; Eira *et al*., 2009; Oyoo-Okoth *et al*., 2010; Baruš *et al*., 2012; Brázová *et al*., 2015; Torres *et al*., 2015; Leite *et al*., 2021) and nematodes (Bergey *et al*., 2002; Hursky and Pietrock, 2015). Similar patterns were reported also for organic pollutants, with acanthocephalan infected fish (Brázová *et al*., 2012) having significant lower concentrations of PCBs. From a theoretical point of view, one would expect less severe effects if the pollutant concentration in infected hosts is lower than in non-infected hosts. Lower pollutant-related toxicity could also result if alternative physiological pathways are activated by parasites, leading to changes in the host's pollutant metabolism. However, these reduced pollutant effects must of course be weighed against the pathological effects of parasites. And this assessment must be carried out individually for each host–parasite–pollutant combination, so that we are currently still a long way from understanding antagonistic effects of parasites and pollutants. Beneficial effects of infection with acanthocephalans on the physiology of fish (reduced oxidative damage) were recorded in natural habitats with higher levels of organic micropollutants (Molbert *et al*., 2020) and in laboratory exposure studies with PAH (Molbert *et al*., 2021). Also from non-vertebrate hosts antagonistic interactions between pollutants and parasites are known. Freshwater mussels, *Pisidium amnicum*, partially infected with larvae of digenean trematodes, were exposed to pentachlorophenol (PCP), resulting in a significantly shorter survival time of the uninfected mussels compared to infected conspecifics, which survived up to twice as long (Heinonen *et al*., 2001). For *Artemia parthenogenetica* Sánchez *et al*. (2016) demonstrated a higher host resistance to increasing arsenic concentrations for intermediate hosts infected with different parasite species.

Compared to additive or synergistic interactions of parasites and pollutants, there have been few studies on antagonistic effects where parasites can reduce pollutant effects. Parasite-reduced pollutant concentrations in infected hosts certainly appear beneficial if hosts face increasing levels of pollution, as lower pollutant concentrations are usually associated with less toxic effects. This relationship should be explored in more detail in future studies, with a clear focus on whether the negative effects of a parasitosis can be outweighed by the potentially positive effects of lower pollutant concentrations. In addressing these aspects, studies should be conducted not only at the individual level but also, if possible, at the population and ecosystem levels.

## Conclusions

The interactions between pollutants and parasites presented here show that parasites must be regarded as organisms that are in close mutual exchange with pollutants. This interaction of parasites and pollutants is significant for both – the occurrence of parasites in ecosystems and for the health of their hosts. In environmental studies, parasites need to be taken into account because they can influence the interaction between fish and pollutants and thus the results of the studies in general. Lower accumulation of contaminants in organism infected with parasites, for example, could make free-living established indicator organisms less reliable for environmental monitoring. On the other hand, contaminants are capable of significantly affecting fish–parasite interactions and parasite transmission, which may lead to an increase or decrease in parasite infestation in fish populations. Systematic studies on the mechanisms of action and the complexity of the interaction between fish and parasites in a polluted environment are largely lacking, so that the basic relationships are often poorly understood. It is known that parasites are also successfully transferred to the next host in polluted environments through host manipulation (e.g. Fanton *et al*., 2020). However, there is not much research studies on changes in host behaviour under pollution conditions and how this affects the transmission efficiency of parasites. Acute or chronic toxic effects on e.g. intermediate hosts may weaken them and make them more susceptible to predation by fish, which could be beneficial for trophically transmitted parasites (e.g. Acanthocephala, Cestoda).
